# What are the mechanisms that support healthcare professionals to adopt assisted decision-making practice? A rapid realist review

**DOI:** 10.1186/s12913-019-4802-x

**Published:** 2019-12-12

**Authors:** Carmel Davies, Francesco Fattori, Deirdre O’Donnell, Sarah Donnelly, Éidín Ní Shé, Marie O. Shea, Lucia Prihodova, Caoimhe Gleeson, Áine Flynn, Bernadette Rock, Jacqueline Grogan, Michelle O’Brien, Shane O’Hanlon, Marie Therese Cooney, Marie Tighe, Thilo Kroll

**Affiliations:** 1 School of Nursing, Midwifery and Health Systems, University College Dublin, Dublin, Ireland; 2 School of Social Policy, Social Work and Social Justice, University College Dublin, Dublin, Ireland; 3 Royal College of Physicians of Ireland, Dublin, Ireland; 4 Health Service Executive, Dublin, Ireland; 5 Decision Support Service, Mental Health Commission, Dublin, Ireland; 6 The Alzheimer Society of Ireland, Dublin, Ireland; 7 Medicine for the Elderly, St. Vincent’s University Hospital, Dublin, Ireland

**Keywords:** Assisted/supported decision-making, Implementation science, Healthcare, Rapid realist review

## Abstract

**Background:**

The United Nations Convention on the Rights of Persons with Disabilities (UNCRPD) establishes a right to legal capacity for all people, including those with support needs. People with disabilities have a legal right to be given the appropriate supports to make informed decisions in all aspects of their lives, including health. In Ireland, the Assisted Decision-Making (Capacity) Act (2015) ratifies the Convention and has established a legal framework for Assisted Decision Making (ADM). The main provisions of the Act are not yet implemented. Codes of Practice to guide health and social care professionals are currently being developed. Internationally, concerns are expressed that ADM implementation is poorly understood. Using realist synthesis, this study aims to identify Programme Theory (PT) that will inform ADM implementation in healthcare.

**Methods:**

A Rapid Realist Review using collaborative methods was chosen to appraise relevant literature and engage knowledge users from Irish health and social care. The review was led by an expert panel of relevant stakeholders that developed the research question which asks, ‘what mechanisms enable healthcare professionals to adopt ADM into practice?’

To ensure the PT was inclusive of local contextual influences, five reference panels were conducted with healthcare professionals, family carers and people with dementia. PT was refined and tested iteratively through knowledge synthesis informed by forty-seven primary studies, reference panel discussions and expert panel refinement and consensus.

**Results:**

The review has developed an explanatory PT on ADM implementation in healthcare practice. The review identified four implementation domains as significant. These are Personalisation of Health & ADM Service Provision, Culture & Leadership, Environmental & Social Re-structuring and Education, Training & Enablement. Each domain is presented as an explanatory PT statement using realist convention that identifies context, mechanism and outcome configurations.

**Conclusions:**

This realist review makes a unique contribution to this field. The PT can be applied by policymakers to inform intervention development and implementation strategy. It informs the imminent policy and practice developments in Ireland and has relevance for other worldwide healthcare systems dealing with similar legislative changes in line with UNCRPD.

## Background

The United Nations Convention on the Rights of Persons with Disabilities (UNCRPD) is a human rights instrument to establish a right to legal capacity for all people, including those with support needs [1]. States that have ratified the Convention are accountable to address existing barriers to this right, in legislation, policies, and practices. It enables people with disabilities a fundamental right to be given the appropriate supports to make informed decisions in all aspects of their lives, including health [2]. The Convention recognises the importance of human autonomy and self-determination [3, 4]. In the context of UNCRPD, the term Supported Decision Making (SDM) is used and has specific legal connotations. This places relevant individuals at the centre of decision-making regarding their lives and emphasises due regard for their will and preferences. SDM also describes the provision of appropriate assistance which maximises the decision-making capacity of a relevant individual. If deemed necessary, individuals can name trusted supporters to assist them [5, 6].

Many countries such as Australia, Canada, Israel, Ireland, Germany, the United Kingdom and some states in the United States of America have ratified the Convention and made the appropriate legislative changes. Various terms are used interchangeably across jurisdictions and in the literature. These include ‘supported decision making’, ‘active decision making’, and ‘assisted decision making’ [2]. It is important to emphasise that terms are not interchangeable if they refer to the legal frameworks available for the person to make decisions within a particular jurisdiction. This paper uses the term ‘Assisted Decision Making’ (ADM) throughout because the review presented was conducted in Ireland and the term ADM reflects the language of the Assisted Decision-Making (Capacity) Act in Ireland [7].

The Assisted Decision-Making (ADM) (Capacity) Act was enacted in Ireland in 2015 [7]. It offers new arrangements, procedures, and structures under its guidance. Under the Act, there is a statutory presumption that all individuals have decision-making capacity. However, capacity is context and time-bound. Functional capacity tests determine capacity within a specific time and for a specific issue and context. The Act provides a statutory framework of tiered decision supports appropriate to the level of decision-making capacity. At the lowest and least formal level of decision making, a person whose decision-making capacity is or may be called into question may appoint a decision-making assistant to help him or her assimilating information and communicating his or her decision. On the next tier, the person may legally appoint a co-decision-maker with whom he or she will make decisions jointly. At the upper level of decision making, the court may make a declaration of incapacity in relation to a specific matter or matters and may appoint a decision-making representative to act as substitute decision-maker. The Act does not stipulate that incapacity must be attributed to a medical cause and could apply to those with intellectual, developmental and psycho-social disabilities. This also includes dementia and acquired brain injuries as well as those with fluctuating capacity. The Act introduces statutory Advance Healthcare Directives (AHD) into Irish law. Furthermore, it mandates for the abolition of the existing system of guardianship arrangements (Wards of Court) and provides for the establishment of the Office of the Decision Support Services which has regulatory and information functions [7].

Within the Irish health system, the legislation offers Healthcare Professionals (HCPs) a legal framework to enable decision making pertaining to the relevant person with their will and preference at the centre of those decisions. The existing National Consent Policy affirms a ‘functional’ definition of decision-making capacity and operates in accordance with common law [8, 9]. ADM Codes of Practice are currently being developed. At the time of writing, there has been no commencement of the main provisions of the Act.

ADM is a complex clinical intervention involving multi-dimensional components that operate across many settings, organisations, professions and care sectors. Disability researcher, Dr. Anna Arstein-Kerslake has emphasised the need for close attention on supported decision-making implementation**.**

*Without close attention to the mechanics of how supported decision-making is implemented, there is a risk that it will become another tick box exercise, more to serve a bureaucratic purpose than to provide genuine choice and control for people with disability* [2].

This paper focuses on how healthcare professionals can be supported to adopt and implement ADM in their practice. Our interest in implementation is significant, given the recognition that many new interventions fail to translate into meaningful patient outcomes. Other autonomy movements, such as ‘Shared Decision Making’ and ‘Person-Centred Practice’ have faced significant implementation challenges [2, 10–12]. The Codes of Practice for the Mental Capacity Act (2005) in the United Kingdom (UK) continue to experience challenges with embedding the codes into routine practice [13–15]. There is substantial literature that indicates ‘context’ is a powerful influence on the integration of new interventions into practice. Contextual influences are often represented as implementation barriers or facilitators. A more nuanced interpretation of context is that it represents the ordinary conditions of practice. It can involve pre-existing structures and processes, historical patterns of relationships, cultural and social norms, politics, economics and behaviours [16–22]. Given the complex and context-dependent nature of implementing change, there is increasing attention on studying context so that more appropriate policies and interventions can be developed to support behaviour change and implementation [23]. There is no robust evidence base that focuses on ADM implementation.

This article describes a Rapid Realist Review (RRR) which outlines four guiding principles to support the implementation of ADM, that takes account of international research and the Irish healthcare policy and practice context. Realist approaches allow us to examine how ADM relates to both the individuals involved and in the context of its implementation within a health system. In this way, it identifies the enabling and constraining dynamics of its implementation [24, 25]. While the findings are of direct value to inform healthcare policymakers and practitioners in Ireland, there is transferrable learning for any jurisdiction operating similar legal frameworks related to supported decision making.

## Method

This review is part of a larger multi-phase Promoting Assisted Decision Making in Acute Care settings (PADMACs) study [25]. Rapid reviews have emerged as a streamlined approach to synthesising evidence, usually for informing emergent decisions faced by policymakers and practitioners in health care settings [26]. A RRR approach was chosen to gather and appraise the relevant literature as it explicitly allows for the engagement of policymakers and knowledge users throughout the review process. This ensures the results are relevant to current practice, making them more useful for implementation planning [27–31]. This review adopts a collaborative model involving an expert panel and follows five iterative stages, which have been applied by others previously [29, 31]. These include:

1. Developing and refining a research question.
2. Searching and retrieving information.
3. Screening information.
4. Appraising information.
5. Synthesising and interpreting information.

**Fig. 1:**
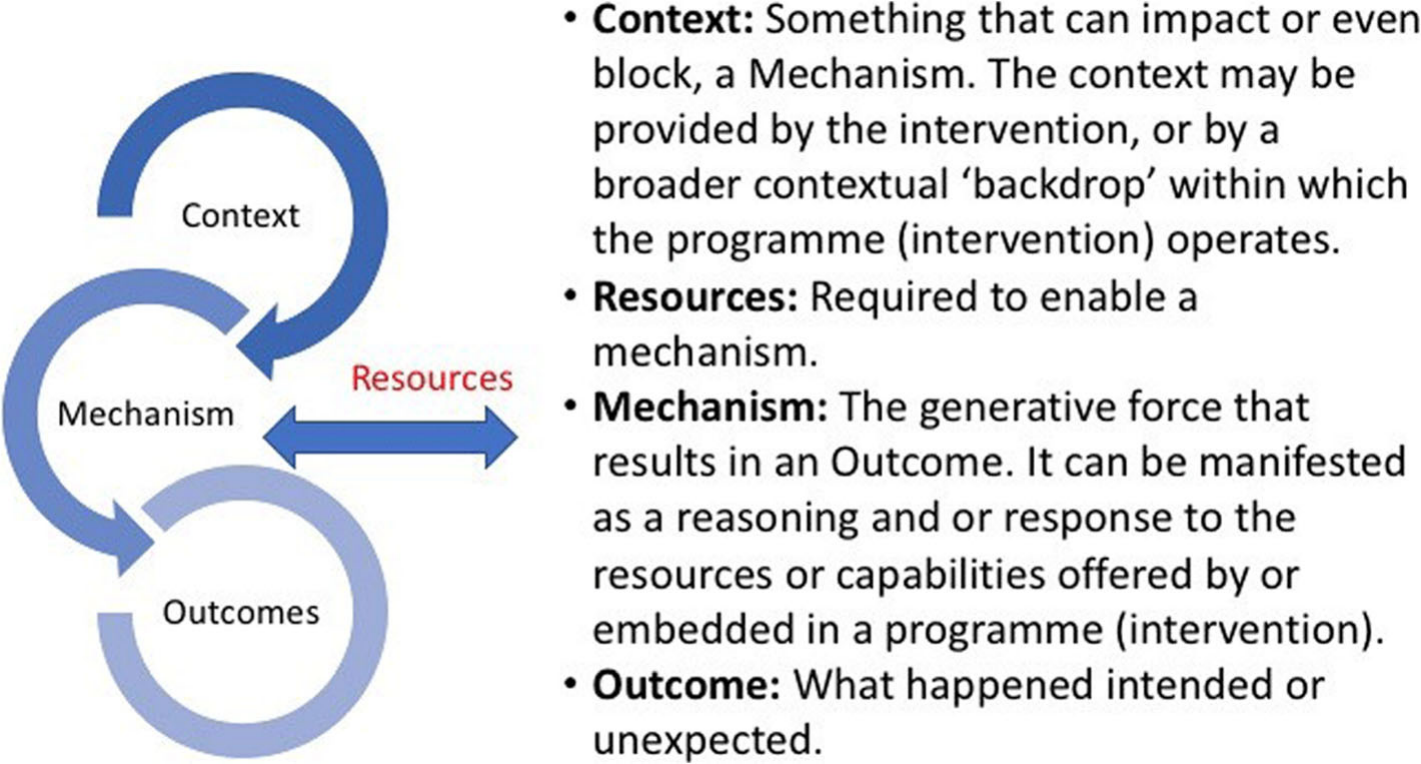
RRR explanation of Context, Mechanism, Outcome and Resource. This image has been reproduced under a CC-BY 4.0 license and gained permission from the author [28]

The units of analysis within realist evaluation are programme theories. These are the ideas and assumptions that describe how, why and in what circumstances complex interventions work. These complexities are unpacked using Context-Mechanism-Outcome (CMO) configurations [24, 32]. Mechanisms are elements that elicit a shift in human reasoning and beliefs and results in a behaviour change outcome [24, 33]. Focusing attention on mechanisms is increasingly recognised as essential because of their significant influence on a change process [33, 34]. Programme theories are statements that connect context, mechanisms and outcomes [32, 35, 36]. They are refined and tested iteratively through knowledge synthesis and expert panel discussions [28]. This review included the identification of resources based on the assumption that specific resources (R) can enable mechanisms (M) and lead to positive changes and outcomes (O) in an existing context (C). Resources were considered as essential to include as they are central to implementation planning. Figure 1 defines the terms and illustrates the CMO (R) relationship [29].

### Establishment of an expert panel

An expert panel was convened in March 2018 consisting of representatives from disability, law, health and social care policy and practice, education and patient advocacy organisations. An additional word file provides details of the expert panel membership (see Additional file 1). All expert panel members are co-authors of this paper. In the first meeting, the expert panel clarified and agreed on the study scope, research question, reference panels [Table 2], literature search strategy and inclusion criteria [Table 3]. A summary of the discussion at the expert panel meeting (March 2018) is available in an additional file [see Additional file 2].

### Scope of the review

An initial phase in a RRR is a discussion between researchers and intended knowledge users to help clarify the scope of the review and prioritise appropriate research questions and objectives. The PADMACs study primarily focuses on assisted decision making with older people within the acute health care setting. This RRR is the first phase of this study. The review was not limited to the acute care setting. It included other healthcare settings to facilitate a broader understanding of the complex nature of ADM. The expert panel agreed that other settings might be more advanced in adopting legislative provisions and implementation efforts.

The review scope was determined by conceptualising the patient care journey. This enabled an examination of contexts, resources, mechanisms and outcomes across the following settings: community/primary care, emergency department, hospital admission, hospital discharge and rehabilitation /residential care. While ADM legislation applies to all individuals, this review focuses on ‘supported’ decision making in persons over 18 years with decision making support needs because of loss of capacity or fluctuating capacity.

### Research question and objectives of the review

The expert panel devised the research question that asks: *what are the mechanisms that support healthcare professionals to adopt assisted decision-making into practice?* The question was set in the context of the introduction of the ADM (Capacity) Act in Ireland [7]. It aims to provide policymakers with some guidance for implementation planning within the health system. The terms of the research question are outlined in Table 1.

**Table 1:** Terms of the research question defined

Term	Definition
Assisted Decision-Making	This term is informed by the Assisted Decision Making (Capacity) Act 2015. Assisted decision-making is understood as operating via two relational pillars: the presumption of capacity afforded to everybody and the necessity to support an individual’s autonomy about every decision to be made about their care. This includes actions to maximise a person’s right to make their own decisions, with legally recognised supports, wherever practicable.
Mechanisms	This term is understood from a realist perspective as part of a Context, Mechanism, Outcome (CMO) configuration. The term mechanism describes a generative force that elicits a shift in the subjects’ reasoning and beliefs and results in a behaviour change outcome [24].

The objectives of the realist synthesis are the following:

• To develop a better understanding of the contextual factors that can contribute to successful ADM in healthcare.

•To identify the mechanisms and resources that are likely to foster ADM implementation in healthcare.

• To develop an explanatory programme theory on ADM implementation that informs early-stage implementation planning across a health system.

### Reference panels

Reference panels are local sounding boards undertaken in an RRR to ensure that the review and programme theory has considered and been informed by local contextual influences and experience of the knowledge user. This reference panel process was guided by a method used previously [28–31]. The purpose of the reference panels was to gain some insights into what the legislation will mean at the local level and ensure that these issues are considered in line with the literature synthesis

The expert panel identified and invited relevant organisations to contribute to the reference panel process. Knowledge users included representation from healthcare professionals, family carers and people with dementia. Five reference panels were conducted from April to November 2018 and were facilitated by expert panel members (CD, DOD, FF, SD, BR, TK and ENS). Table 2 outlines the details of the reference panels conducted for the review. Each organisation provided written consent to participate in the realist review. Participants provided verbal consent. The ASI provided grey literature that had been gathered as part of their ADM policy consultation process. Within the reference panels conducted by the researchers, observational notes of key themes were recorded. The summary notes were reviewed and validated by each organisation. The reference panels were not designed to generate generalisable conclusions but rather seek to understand some of the varied perspectives among knowledge users.

**Table 2:** Summary of the Reference Panel

Organisation/role	Reference panel
• National Rehabilitation Hospital, Dublin, Ireland. Tertiary specialist rehabilitation service.	Workshop with the rehabilitation HCP team where they shared the experience of ADM with people that have an acquired cognitive and/or physical disability.
• St. Vincent’s University Hospital, (SVUH). Ireland East Hospital Group. • Mater Misericordiae University Hospital (MMUH). Ireland East Hospital Group. Level four; academic teaching hospitals delivering national and regional acute, chronic and specialist care services.	Workshops held in SVUH and MMUH with the multidisciplinary team for an older person in the acute care setting. HCPs provided their experiences and perspective on the implementation of ADM within the acute care setting.
• Family Carers Ireland (FCI) Advocacy organisation providing service and support for family carers.	A public consultation workshop with members of Family Carers Ireland addressing issues of importance concerning the ADM Act (2015).
• The Alzheimer Society of Ireland (ASI) Advocacy organisation providing dementia-specific services and supports and advocating for the rights and needs of all people living with dementia and their carers.	Grey literature provided from the ASI based on a consultation led by ASI with people living with dementia eliciting their views on what ADM means for them.

### Search strategy procedure

Assisted Decision Making was the primary outcome of interest in its broadest interpretation. The review included any study which focused on shared decision-making, assisted or supported decision making or advanced care planning involving HCPs and patients with impaired or fluctuating capacity. It included studies that consider impaired decision-making capacity temporarily or indefinitely in conditions of dementia, delirium; neuro-cognitive conditions (mental health, intellectual disability), communication impairment and those with impaired capacity at the end of life. Papers that focused on decision making between HCPs and surrogate decision-makers or supporters were also included.

In consultation with the expert panel and based on the research question, the review search terms and inclusion criteria were agreed (Table 3). The search and inclusion criteria were applied to the electronic databases Pubmed, CINAHL and PsycINFO. Expert and reference panel members were invited to contribute other articles they deemed relevant for the study.

**Table 3:** RRR literature search strategy and inclusion criteria

Database Search:	Pubmed, CINAHL and PsycINFO
String 1. Participants:	Doctor, Physician, Social Work, Health Personnel, General Practitioner, Dietitian, Radiographer, Radiologist, Occupational Therapist, Physiotherapist, Speech & Language Therapist, Social Worker, Psychologist, Nurse, Midwife
String 2 Outcomes	Shared Decision-Making, Patient Participation, Advance Care Planning, Advance Care Directives, Mental Competency, Living Wills, Patient Care Plans, Assisted Decision-Making, Supported Decision-Making, Patient Decision-Making, Nursing Care Plans, Family Decision-Making Keywords, MESH and headings were identified by the expert panel under participant and outcome search strings and combined using Boolean operators.
Inclusion/exclusion criteria	Quantitative and qualitative peer-reviewed articles, English language, 10-year timeframe (2008–2018), Human subject older than 18 years. Round two and three inclusion /exclusion criteria are available in additional file 3.

### Screening and appraisal

**Fig. 2:**
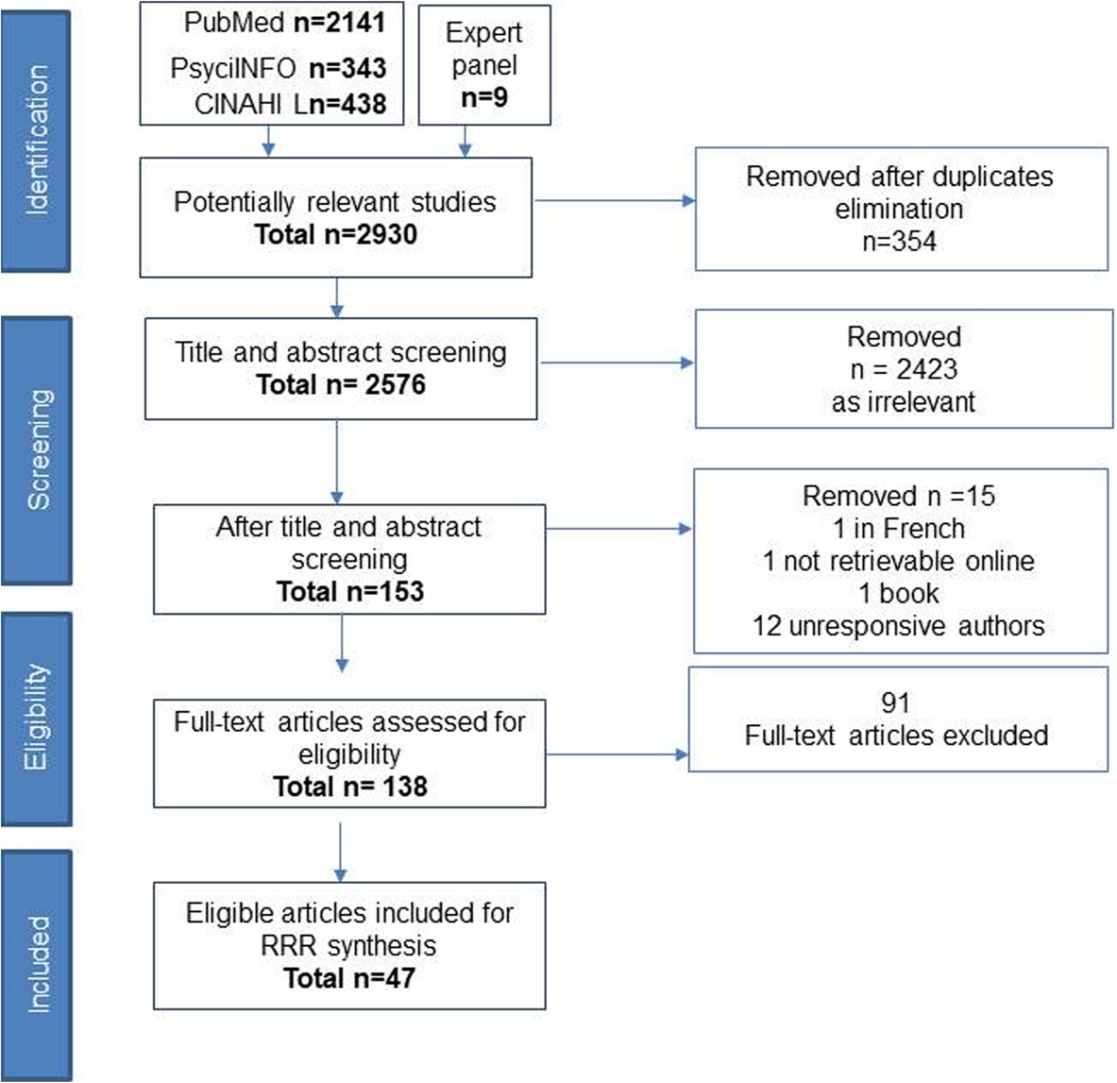
Adapted Prisma flow chart

The data screening involved a three-stage process. Figure 2 represents this process with an adapted PRISMA flow diagram. After the elimination of duplicate papers, a total of 2576 papers were identified for screening. Five authors (CD, FF, DOD, SD and ENS) conducted screening of title and abstract. Irrelevant studies were eliminated. This included papers that only measured the prevalence of shared decision making in different health contexts, health literacy as opposed to competency, participation in clinical trials and validation studies of psychometric scales for measuring shared decision making. In line with realist approaches, the primary inclusion /exclusion criteria were refined to ensure closer alignment with the scope of the research question. Further details on the refined inclusion criteria are available in an additional file (see Additional file 3).

Stage two of the process involved full-text review on 138 papers (CD, FF, DOD, SD and ENS). Weekly consensus meetings were held to conduct inter-rater reliability testing. Any differences of opinion about study eligibility were discussed, and a consensus reached. The process of screening was completed in August 2018. Forty-seven papers were eligible for review synthesis.

### Literature data extraction

Full-text extraction and synthesis of the primary literature were based on 47 documents. At the first expert panel meeting, it was agreed that programme theories should be practical and relevant to guide ADM implementation within the health system. The expert panel created an extraction template which facilitated that CMOs were mapped to the Behaviour Change Wheel (BCW) framework proposed by Michie et al. [37, 38]. The BCW is a recent but increasingly popular taxonomy to assist in implementation planning. The review linked to the BCW policy categories and intervention functions which connected the programme theories to an evidence-based framework that focuses on behaviour change and appropriate for implementing change.

This data extraction tool was adapted based on previous appraisal work within a rapid realist project [31]. The tool allows papers to be appraised for rigour and relevance. Evidence was not excluded based on this appraisal, but focused on conceptually rich papers, without excluding weaker papers which may still contribute within realist methodologies to the final evidence synthesis [31]. Final data extraction and analysis was undertaken by the synthesis leads (CD, FF) and validated by some expert panel members (DOD, SD, ENS, TK) in weekly consensus meetings. The extraction template and further detail on the Behaviour Change Wheel (BCW) framework are available in an additional file (see Additional file 4).

### Nature of the data set

The PT was informed by grey literature from five reference panels (Table 2) and 47 research documents. The summary notes from the reference panels are available in an additional file (see Additional file 5). A summary of all the literature documents supporting the review is available in an additional file [see Additional file 6]. The primary studies included 38 papers from the data search and nine from the expert panel. The papers were a mix of intervention (*n* = 7) and descriptive studies (*n* = 40). The intervention studies measured patient-centred outcomes rather than assisted decision-making effectiveness or implementation. The descriptive studies included qualitative and quantitative cross-sectional survey designs.

The studies predominantly focused on single components of assisted decision making. The topics of predominance in the review were advanced care planning, shared decision making, advance care directives and proxy decision making. Interventions studies focused on decision aids (n-6) and a dementia support consultation service (*n* = 1). The studies included were mainly from developed countries including UK (*n* = 7), Germany (*n* = 3), Switzerland (*n* = 3), Belgium (*n* = 3), Norway (*n* = 3), Ireland (*n* = 2), Netherlands (*n* = 1), Finland (*n* = 1), Italy (*n* = 1), Israel (*n* = 1), United States (*n* = 14), Canada (*n* = 3), Brazil (*n* = 1) and Australia (*n* = 4).

The empirical findings helped to understand the existing contextual factors operating within ADM practices and participant’s responses reflected their underlying reasoning and motivations (mechanisms) in response to ADM interventions. The reference panels added valuable contextual knowledge from “on the ground” experiences and realities in light of the emerging ADM (Capacity) Act in Ireland [7].

### Programme theory (PT) process

The process of generating PT was an iterative process using data extracted from reference panels (grey literature), research literature and refined by the expert panel. The first phase involved a summary of the key themes from the reference panels (DOD, CD, SD, ENS and FF). These were drafted into nine Initial Programme Theories (IPTs). A more detailed overview of this process is provided in an additional file [see Additional file 5]. The literature analysis refined the IPTs into four distinct but related principles that formed the final PT. This was presented to the expert panel with a discussion of the supporting evidence. The expert panel refined the wording of the Programme Theory CMO statements and generated consensus on the resources required. Some additional review papers from Implementation Science were included from the expert panel to help refine the programme theory on ‘Culture & Leadership’ as this was not represented within the ADM literature. This flexibility is in line with the RRR methodology [28]. There is an additional file representing the summary notes of each process of programme theory development. This additional file includes the reference panel notes, initial programme theories and the notes from the final expert panel meeting (see Additional file 5). The PT was finalised in November 2018.

## Results

**Fig. 3:**
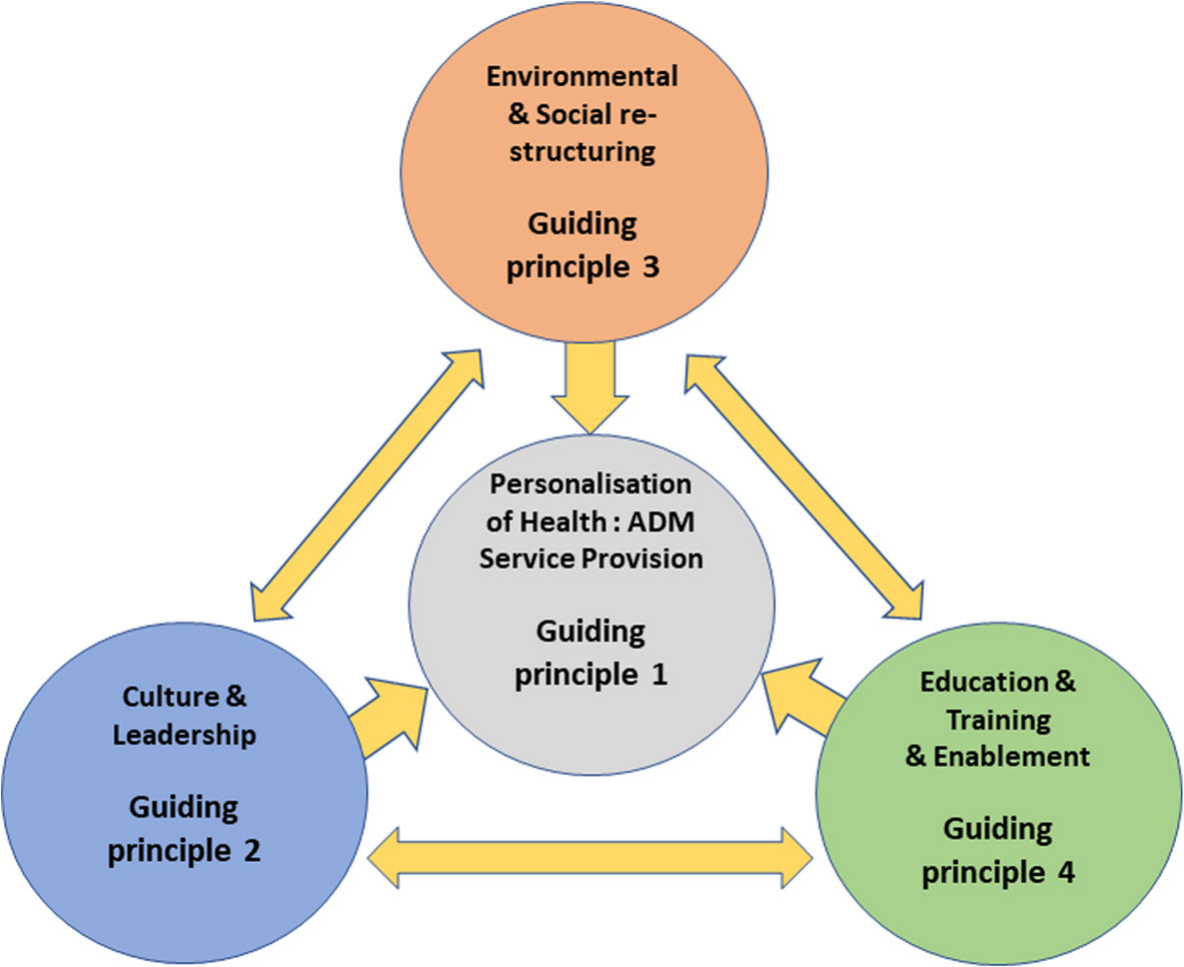
Programme Theory Implementation domains for Assisted Decision Making in Healthcare. Programme Theory represented as Implementation Domains

The PT entitled ‘Assisted Decision-Making Implementation in Healthcare Practice’ is presented as four guiding principles with CMO and resource explanatory statements. The programme theory is mapped to implementation domains which are represented in Fig. 3. While each domain is presented separately, figure three illustrates how they are inter-related and interdependent. Personalisation of Health & ADM Service Provision highlights the need to implement ADM through a formal service within a health system. Embedding a new service into a health system is challenging and involves radical organisational reform. Implementation support is critical, and the review identified ‘Culture and Leadership’, Environmental and Social Re-structuring and Education, Training & Enablement as key priorities for an implementation strategy. The programme theory explanatory CMO statements are presented for each domain in Table 4 below.

**Table 4:** Programme Theory (PT) Assisted Decision-Making Implementation in Healthcare Practice

**Guiding principle 1 Personalisation of Health - ADM Service Provision**	
Health systems that embed ADM as a core principle of person-centred care (C), into a formal service (R), normalise care planning practice (M) and maximise individuals’ autonomy and capacity to participate in decisions about their health and care (O).	
**Guiding principle 2 Culture & Leadership**	
Health Systems where ADM is a prioritised shared vision (C), influence positive ADM culture (M), and when enabled by senior leadership and an adequately funded implementation strategy (R), generates commitment, accountability, and engagement across the whole organisation (M) facilitating ADM into practice (O).	
**Guiding principle 3 Environmental & Social Re-structuring**	
Healthcare Systems where their social and physical environment enable assisted decision making through interdisciplinary person-centred practice (C) provide appropriate support (R), which augments an individual’s trust and confidence (M) and maximises their autonomy and capacity to participate in decisions about their health and care (O).	
**Guiding principle 4 Education, Training & Enablement**	
Health systems that have a strong learning culture (C) and invest in public guidance and staff interdisciplinary education and training (R) foster positive knowledge, attitudes and skills (M) leading to confident practitioners and an empowered public that engages in ADM (O).	

### Guiding principles that informed the Programme theory

The following guiding principles informed the PT presented in Table 4. They were informed by knowledge synthesis and expert panel discussions and should not be interpreted as guiding principles from the ADM (Capacity) Act (2015) or related Codes of Practice.

#### Guiding principle 1: personalisation of health & ADM service provision

Supporting the translation of the ADM (Capacity) Act (2015) into healthcare requires interventions that are driven by an agenda that focuses on patient needs [16]. In terms of ADM, this requires efforts and commitment to support and protect the autonomy of the person with a cognitive disability in all decisions related to their health and social care [2]. The expert panel identified ADM as a core principle of person-centred care. Many studies, including one from Ireland, highlighted several contextual influences that mitigate against the provision of quality person-centred care in relation to ADM. These include lack of time, competing clinical work, fragmented care services, inadequate professional collaboration and uncertainty in professional roles in relation to ADM. The review identified ADM as a complex intervention and frequently unpredictable in nature [39–55].

The need for a formalised approach to care planning was a strong recommendation across the literature. The concept of ADM Care Planning is not an inherent part of the ADM (Capacity) Act 2015. However, it may offer HCPs a better opportunity for meaningful and quality engagement in the ADM process. While the expert panel viewed ADM as the business of all HCPs, they felt that a care plan service would provide a scaffold to formalise ADM implementation within a healthcare system. Many studies used the term ‘Advanced’ Care Planning’ (ACP) to indicate planning for the future. However, the expert panel advocated for the term ‘care planning’ as its scope is more inclusive and not restricted to end of life care. Care planning is described as a ‘better conversation’ that helps people with long-term conditions to be in control of planning their care [34]. Care planning specific to ADM ensures that the relevant individual is always central in the decision-making network. It is ideal when it operates within an integrated and interdisciplinary person-centred care model. This is particularly important for consistency in care planning for those with complex needs that utilise several health care services [39–45].

Several other issues were identified in the literature that is relevant for programme developers seeking to establish ADM within healthcare [41–43, 46–51]. ADM is enhanced by early engagement in the process. This maximises the time required to explore and identify an individual’s values and care goals. Early engagement also informs individuals about the formal (legal) aspects of ADM, so they have a clear expectation of their rights. Eliciting an individual’s values and preferences early is crucial for informing more formal processes such as ACP and AHD. Family members identified these issues as a promising way to minimise tensions that often emerge between family and healthcare professionals and the burden associated with existing models of substituted decision making [52]. Engagement in care planning requires a formal assessment to identify an individual’s readiness to engage, as this has been found to vary significantly for older people [53]. Transitioning to care planning is also often affected by the patient and relative’s denial of a diagnosis. This was highlighted in a study of patients with a recent dementia diagnosis [54]. Therefore, an ADM care plan service should embed the opportunity for the provision of targeted supports to help people understand and cope with their diagnosis.

Across the studies, it was evident that patients see ADM quality as very much linked with the process of relationship building. Making time to build a therapeutic relationship and to ‘get to know the person’ as well as communicating in a person-centred style is critical [55]. These conditions help generate a sense of having a ‘trusted ally’ which augments an individual’s sense of safety and confidence to engage in making decisions [56]. Decision aids should be accessible to all individuals needing decision support. Several studies identified that their value should be dictated by the individual requiring support rather than proven efficacy or HCP preference [55, 57, 58].

The guiding principle of *Personalisation of Health & ADM Service Provision* highlights the value of formal service to assist ADM implementation. ADM should also be considered a core principle of person-centred care. The context of a person-centred care service that focuses on care planning seems to generate a favourable climate that, in turn, generates positive mechanisms and successful outcomes (Table 4 provides the summary PT).

#### Guiding principle 2: culture and leadership

The reference and expert panel discussions strongly emphasised the need for system-wide commitment through culture and leadership. There was less direct attention placed on this topic within the ADM literature. Some supplementary literature from Implementation Science helped to refine this programme theory. A recent systematic review identified organisational culture and leadership as critical features that influence implementation practice across healthcare settings [21].

The starting point for healthcare organisations is to establish a shared vision [16]. This should reflect patient needs, followed by a visible commitment to remove any existing barriers that might hinder the process. A shared vision shapes an organisation’s culture by setting out priorities for service provision. It defines the behaviours expected of HCPs that work within the organisation. The expert panel stressed that ADM must be every HCP’s business.

A shared vision for ADM can only be operationalised effectively with a robust ADM implementation strategy and sufficient resources (human and fiscal). Senior leadership play a crucial role in driving change through the allocation of resources, implementation policies and the development of ADM clinical guidelines. It is also vital for creating an implementation climate that is amenable to integrating a new programme [16]. Organisations that fail to provide leadership and resources generate excessive burden on front line HCPs and increase the likelihood of implementation failure [59]. Senior leadership engagement is essential as it fosters middle manager commitment to act as champions to facilitate the change process. They, in turn, ensure that frontline staff are committed and engaged in the change process, while also generating opportunity for communication between front line staff and senior management [59, 60].

The culture and leadership principle emphasises a shared vision as the basis for a positive ADM culture. When supported by leadership and resources, it can foster commitment, accountability and engagement across the whole organisation. These mechanisms are well recognised as the optimal conditions to enact new forms of practice [16].

#### Guiding principle 3: environmental and social re-structuring

Implementing ADM into practice requires healthcare organisations to conduct assessments of their environment and to identify and remove any barriers (physical and social) to ADM. This may require the restructuring of the healthcare environment to ensure that a hospitable environment for ADM exists. HCPs identified clinical guidelines as a supportive mechanism to enable social planning and clinical guidance for the practice changes required [40, 61].

A key barrier identified within the review related to competing work demands within pressurised healthcare settings. This was a key concern for HCPs working in acute care settings. HCPs from the rehabilitation reference panel presented ADM case examples which emphasised the time-intensive nature of the practice. Staff resources and operationalising caseload capacity are, therefore critical considerations if setting up a formal ADM service [39, 42].

Lack of inter-professional collaboration and ‘perceived’ professional isolation was identified as a key challenge that might impact negatively on ADM. The literature showed that medical practitioners predominantly took responsibility for ADM and identified the potential of a resource model that widens the involvement of interdisciplinary teams [42, 49, 62, 63]. Positive interdisciplinary working relationships were identified across all the HCP reference panels as a supportive mechanism for ADM implementation. Team approaches can also foster positive collaborative relations, mutual support, organisational learning and drive continuous cycles of practice improvement [17, 42, 62].

Another critical issue raised within the literature was the need to ensure that the environment is hospitable to enable good communication and that the individual is provided with whatever supports they find useful. Communication modes include verbal, writing, visual aids, sign language and assistive technology. Several digital devices to support communication and decision making exist including Augmentative and Alternative Communication (AAC), eye gazing devices, spelling boards and other assistive technologies [46, 55, 57, 63, 64]. Many of the studies indicated that a private physical space is important to foster a quality clinical-patient consultation [39, 44, 45].

Care planning is more efficient and effective when information can be shared electronically across a health system. A US-based advanced dementia consultation service attributed programme success to effective information sharing across their health services [49]. The lack of a national electronic health recording system is a significant barrier to integrated ADM care planning [45, 61]. Health policymakers should collaborate and assess what is feasible within their health system to maximise ways to provide consistent information sharing that would enhance care for those requiring support with decision making and care planning.

This guiding principle focuses on the need for organisations to invest in their environment from a social and physical perspective and plan to remove existing barriers to ADM. This will foster a more hospitable and supporting environment for both the relevant individual and HCP (Table 4 provides the summary PT).

#### Guiding principle 4: education, training & enablement

The need for education and training in ADM was a significant finding in this review. Health systems with a strong learning culture foster a better climate to enable ADM practice. Innovative education and authentic learning facilitated through mentorship, and inter-professional peer support is essential mechanisms to foster better ADM practice [57, 65]. It provides the contextual conditions for HCP behaviour change for evidence-based practices [21]. Cartwright [66] found that advanced legal knowledge and excellent communication skills between physician, patient, and family can reduce conflict and the risk of legal challenges. Education and training should include legal and ethical content [42, 45]. Ensuring that healthcare professionals better understand dementia and its implications for decision making was a consistent concern for people with dementia [43, 55, 67, 68]. The inclusion of skills-based training in person-centred communication was cited several times. Poor communication skills were deemed detrimental to the ADM process [41, 56, 64, 69].

Public awareness campaigns are essential to help foster public engagement on the issue [65, 70]. The availability of supporting documents and guidance value statements for the public is warranted. Programmes that are designed to empower people to engage should be co-designed and draw on other evidence-informed programmes such as *Think Ahead* and *Keep Control* [71, 72]. The members of the expert panel involved in health and social care professional education emphasised the need to embed ADM in both undergraduate (pre-registration), postgraduate and Continuing Professional Development programmes. Education and training programmes should be inter-professional and include models for mentoring and training within the practice setting. Patients and the public should contribute to curriculum planning and have a role in delivering ADM education and training.

Investment in education will enhance professional competence and promote commitment, motivation, and confidence in ADM practice [16, 21, 62, 73]. Trained clinicians and informed patients and families will have more productive relationships and increase the likelihood of successful ADM (Table 4 provides the summary PT).

## Discussion

Legislative frameworks for assisted/supported decision-making are present in several countries around the world [1]. Health professionals are accountable to ensure that a person with support needs have all the appropriate supports to assist them in their decision making [2]. It is incumbent on policymakers to ensure that HCPs are adequately supported to adopt ADM into their practice. Wider implementation literature acknowledges that HCPs can feel overburdened if they are expected to adopt new practices in an environment that is not adequately resourced or supportive for a change in practice [17, 59, 73].

### Summary of findings

This RRR identified a programme theory of four inter-related guiding principles that might best support HCPs to adopt ADM into practice efficiently and effectively. These are [1] Personalisation of Health & ADM Service Provision [2] Environmental and Social Re-structuring [3] Culture and Leadership and [4] Education, Training & Enablement. Guiding principle one focuses on ADM as a formal service provision and the remaining three describe strategies that can support the service. The review highlighted the elements of context that can affect how these guiding principles are operationalised and provides some explanation of the mechanisms by which they affect human behaviour and thereby influence change.

The review identified the complex, multi-dimensional and unpredictable reality of ADM. It emphasised the need to embed ADM as a core principle of person-centred care within a formal care plan service. This recommendation was considered a plausible solution to overcome the common contextual barriers that mitigate its success in clinical practice. Contextual barriers identified include lack of adequate time, competing work demands, fragmented care services and isolated healthcare professionals [39–55]. The need to ensure ADM is given a dedicated consultation enables time for therapeutic relationship building between the patient and HCP. This, in turn, generates trust and enables a person to feel more confident engaging in ADM [59, 60].

The need for health system re-structuring was another key finding within the review that considered the reconfiguration of social processes or environmental structures. The most consistent recommendation was the need to scaffold an ADM service within a supportive interdisciplinary team-based approach. Interdisciplinary models of care foster better collaborative relationships among HCPs, enhancing the opportunity for mentoring and support.

The review identified culture & leadership as critical to foster a system-level commitment to the implementation of ADM. A strong shared vision accompanied by an implementation plan and adequate resources fosters system-level commitment [16, 21, 63]. The significance of a shared vision is noteworthy. When the vision is led by senior leadership, it combats the risk of focusing too early on task, process and operational issues which often generate preventable implementation challenges [16]. It also fosters senior management and front-line staff to become agents in the change process generating mechanisms of commitment and collective action. These mechanisms are recognised within the broader literature as critical to successful implementation [21, 73, 74].

Investing in developing personal capacity and competence in ADM through education and training was identified as a critical requirement to support HCPs [21, 39, 40, 49, 59, 67, 69, 70]. Innovative mentorship and inter-professional forums were highlighted as the most progressive way to enable learning that can inform practice. Organisations that foster a strong learning culture accelerate change as the clinician’s knowledge becomes assimilated into new work structures, routines, and norms within an organisation [75]. Health systems with a strong learning culture need to invest in ADM public communication and marketing campaigns to empower public engagement and action in the process.

### Contribution of this review to the existing evidence base

Mapping the CMOs to the Behaviour Change Wheel highlights the range of implementation interventions and policy initiatives recommended in the review. Policy initiatives include service provision, social and environmental planning, guideline formation and communication /marketing. These policies prompt practice changes through supporting interventions that target education & training and environmental re-structuring [38]. The policies and interventions we identified are supported by middle-range theory from implementation and behavioural science literature [16, 22, 38].

To the best of our knowledge, this is the first review of its kind as there is no existing theory that provides guiding principles on ADM implementation. There were no evaluation studies on ADM implementation available for this review. This limits our ability to interpret these results against other national and international evidence. The work, therefore, makes a significant contribution to the field. The range of contextual influences documented in this review is relatively similar to other healthcare implementation studies in shared decision making, person-centred care [10–12] and the Mental Capacity Act (UK) [13–15].

#### Guidance on the application of this programme theory

This programme theory is a catalyst for ADM implementation planning within healthcare. Implementation of new interventions is context-dependent; therefore, what works in one context may not work in the same way in another. The framework is intended to act as a visionary framework that is flexible and not overly prescriptive. Modern health systems foster better implementation success using ‘simple rules’ that offer a degree of flexibility in the way they respond to policy changes and new interventions [20, 22]. We recommend that the framework is used in conjunction with a pre-implementation assessment that explores local contextual issues. The framework can then act as a guide to develop appropriate resources that meet the needs of the setting intended [16].

#### Implications for practice and policy

The results of this review have relevance for government policymakers, healthcare organisations, health professionals, educators, researchers and patient associations. The issues from the literature and the reference panels were relatively similar. This highlights the relevance of these results for informing implementation planning for the emerging Codes of Practice across health and social care services in Ireland. It guides healthcare educators to collaborate and develop interdisciplinary ADM education programmes. The PADMACs study is developing an ADM card-based educational tool that will be useful for interprofessional healthcare education. The active involvement of senior policymakers from the ADM Division Support Office and Health Service Executive ADM National Programme will help to accelerate the dissemination of these results to the relevant national policy and practice networks.

This work can also guide other countries undergoing legislative changes in line with the UNCRPD. It has to some degree, addressed concerns expressed by disability law expert Anna Arstein-Kerslake on the lack of attention to date on supported decision making implementation and the risk that it becomes a bureaucratic ‘tick box’ exercise [2]. This framework places central emphasis on supported decision making within a person-centred service which fosters genuine choice and control for people. Realist evaluation is warranted at any point in the implementation of a policy or intervention. This RRR provides methodological guidance that can be applied by others in health service practice or research to conduct similar work to address other complex change efforts within the health and social care setting.

#### Recommendations for further research

The evidence base on assisted/ supported decision-making implementation is in its infancy. Further research is needed to help expand our understanding of the dynamic complexities of ADM. In phase two of the PADMACs study, this PT will be applied to the acute healthcare setting. In-depth qualitative interviews will be held with healthcare professionals and older people on their views of ADM in the acute care setting. The data will be analysed using this PT as a coding framework to test and refine the PT for application in the acute care setting. We recommend that other researchers apply and test this programme theory in a similar way to other social and healthcare settings.

Advanced research in the field could consider prospective comparative case studies using common measures and terminology which facilitate investigating how similar implementation interventions work across varying contexts. While this review has not examined the interactions between the four guiding principles, they appear interrelated. Future research could examine these relationships and the influence interacting mechanisms have on implementation outcomes.

### Strengths and limitations of this review

Unlike systematic reviews which control contexts, realist reviews embrace contextual complexity and provide a better understanding of why and how things work [24]. This approach is well suited to healthcare implementation, which recognises that both the context and intervention influence each other [16]. A strength of the rapid review is the ability to engage directly with policymakers and knowledge users to ensure research questions are relevant and in line with healthcare policy and practice priorities. For policymakers, it provides a practical, relevant outcomes-focused knowledge synthesis informed by both published/research and local/practice-based evidence.

While this review was systematic, it was not exhaustive. We did streamline the review in a deliberate effort to be able to provide policymakers with results in a timely manner. For practical reasons we limited the number of reference panels and could potentially have had more. The review did not include an extensive search for grey literature, and we limited our review to using three databases and English papers. Implementation is so context-dependent that generalisability of these results is to an extent limited. The CMO configurations identified were not clearly defined in the literature and we acknowledge that these relationships are to an extent theorised and need further testing.

## Conclusion

The findings from this review help to focus attention on how those working within complex health systems and organisations can practically support legislative changes and ADM into healthcare practice. This review revealed key considerations for supporting HCPs in adopting ADM. Particularly; systematic implementation planning, and evaluations are needed. The PTs presented here may guide this work. This necessitates close collaboration among policymakers; educators, practice leaders, advocacy groups and patients.

## Supplementary information

**Additional file 1.** Expert Panel Membership.

**Additional file 2.** Expert Panel (March 2018) Summary notes.

**Additional file 3.** Rapid Realist Review: Literature screening process.

**Additional file 4.** Literature Synthesis Extract Template.

**Additional file 5.** Programme Theories (PTs) Process- Phase 1-3.

**Additional file 6.** Summary of literature for Review.

## Data Availability

The templates and datasets for analysis used during the current study are available from the corresponding author on reasonable request.

## Abbreviations

ACP: Advance care planning
ADM (Capacity) Act: Assisted Decision-Making (ADM) (Capacity) Act
ADM: Assisted decision making
AHD: Advance Healthcare Directives
ASI: Alzheimer Society of Ireland
BCW: Behaviour Change Wheel
CMO: Context-Mechanism-Outcome
FCI: Family Carers Ireland
HCPs: Healthcare Professionals
IPTs: Initial programme theories
PT: Programme theory
RRR: Rapid realist review
SDM: Supported decision making
UNCRPD: United Nations Convention on the Rights of Persons with Disabilities
