# Supplementary material for: What are the mechanisms that support healthcare professionals to adopt assisted decision-making practice? A rapid realist review
Source: BMC Health Serv Res. 2019 Dec 12;19:960. doi: 10.1186/s12913-019-4802-x (PMC6909502; doi:10.1186/s12913-019-4802-x)
Supplement: Supplementary file 1 — Additional file 1. Expert Panel Membership. [file 12913_2019_4802_MOESM1_ESM.docx]

**Additional file 1 Expert Panel Membership**

- Dr Deirdre O’Donnell (Chair) , Lecturer, School of Nursing, Midwifery and Health Systems, University College Dublin, Ireland.
- Dr Carmel Davies, Lecturer, School of Nursing, Midwifery and Health Systems, University College Dublin, Ireland.
- Dr Francesco Fattori, Researcher, School of Nursing, Midwifery and Health Systems, University College Dublin, Ireland.
- Dr Sarah Donnelly, Lecturer, School of Social Policy, Social Work and Social Justice, University College Dublin, Ireland.
- Dr Éidín Ní Shé, Researcher, School of Nursing, Midwifery and Health Systems, University College Dublin, Ireland.
- Dr Lucia Prihodova, Research Manager, Royal College of Physicians of Ireland, Dublin, Ireland.
- Ms Caoimhe Gleeson, National Programme Lead- Assisted Decision Making, Health Service Executive, Ireland.
- Ms Áine Flynn, Director of the Decision Support Service, Mental Health Commission Ireland.
- Dr Bernadette Rock, Policy & Research Manager, Alzheimer’s Society of Ireland.
- Ms Jacqueline Grogan, Quality Improvement Division, Health Service Executive, Ireland.
- Dr Michelle O’Brien, Specialist Registrar, Medicine for the Elderly, St. Vincent’s University Hospital, Ireland.
- Dr Shane O'Hanlon, Consultant, Medicine for the Elderly, St. Vincent’s University Hospital, Dublin, Ireland.
- Dr Marie Therese Cooney, Consultant, Medicine for the Elderly, St. Vincent’s University Hospital, Dublin, Ireland.
- Ms Marie Tighe, Quality Improvement Division, Health Service Executive, Ireland.
- Prof Thilo Kroll, Professor of Health Systems Management, School of Nursing, Midwifery and Health Systems, University College Dublin, Ireland.
