# Supplementary material for: What are the mechanisms that support healthcare professionals to adopt assisted decision-making practice? A rapid realist review
Source: BMC Health Serv Res. 2019 Dec 12;19:960. doi: 10.1186/s12913-019-4802-x (PMC6909502; doi:10.1186/s12913-019-4802-x)
Supplement: Supplementary file 2 — Additional file 2. Expert Panel (March 2018) Summary notes. [file 12913_2019_4802_MOESM2_ESM.docx]

**Additional file 2: Expert panel (March 2018)**

**Summary notes**

| **Item** | **Discussion points and agreements** |
| --- | --- |
| **Defining the rapid realist review scope** | The group agreed that healthcare settings outside of hospitals were more likely to have a better understanding of supporting decision-making and were more likely to have already adopted some practices about implementing some of the legislative provisions.  It was agreed not to limit the scope of the rapid realist review to acute care settings but healthcare contexts more generally.  The group came to a consensus that the patient's care journey should provide the guide to the search for evidence as well as the structuring of the subsequent review. This would enable an examination of contexts, resources, mechanisms and outcomes across the following settings: community/primary care, emergency department, hospital admission, hospital discharge and rehabilitation /residential care. |
| **Defining the research questions** | “What are the mechanisms that support healthcare staff to adopt ADM practice?” |
| **Defining**  **Mechanism**  **& resources** | RRR, ‘mechanism’ is understood as an action/intervention which targets behaviours and reasoning and not solely knowledge or skills.  A distinction was made between mechanism and resources. Resources are the physical/structural/social asset which emerges from the context and which is leveraged to enhance a mechanism. The team discussed the value of including resources within the review as they are central in implementation planning. This will be included in the review analysis. |
| **Defining ADM practice** | The expert team agreed that the legislation itself should be the guiding force in understanding the specifics of ‘assisted decision-making’ with particular reference to the functional test for decision-making capacity.  Assisted decision-making should be understood as operating via two relational pillars: the presumption of capacity afforded to everybody and the necessity to exhaust an individual’s autonomy about *every* decision to be made about their care. It was agreed that while ADM practice might remain within the RRR research question, it will have to be defined clearly and operationalised as per the legislation in the extraction of data from the retrieved articles as well as in the realist review of the evidence. |
| **Formulating a purpose statement** | The rapid realist review purpose is to deepen the understanding of the mechanisms which support healthcare staff to adopt assisted decision-making practice when developing and implementing care plans with their patients. National and international descriptive and evaluative evidence inform the review and analysis will involve a realist synthesis of contexts, resources, mechanisms and outcomes. This, in turn, will generate a body of knowledge relevant to the successful implementation of behaviour change about assisted decision-making in healthcare settings. |
| **Developing the search strategy** | Assisted Decision Making was the primary outcome of interest in its broadest interpretation. The review included any study which focused on shared decision-making, assisted or supported decision making or advanced care planning involving HCPs and patients with impaired or fluctuating capacity. It included studies that consider impaired decision-making capacity temporarily or indefinitely in conditions of dementia, delirium; neuro-cognitive conditions (mental health, intellectual disability), communication impairment and those with impaired capacity at the end of life. Papers that focused on decision making between HCPs and surrogate decision-makers or supporters were also included. |
| **Developing the reference panels** | The expert panel agreed that reference panels should be included in the review to ensure that the research (literature) considers tacit contextual experiences of knowledge users that will in future use ADM legislation.  The reference panels were chosen to ensure they provided a range of perspectives from knowledge users (patients, carers and healthcare professionals).  It was agreed that one consultation per panel was deemed sufficient within the short timeframe of the review. Note-taking rather than formal data collection methods were agreed. Data saturation or achieving representative conclusions were not the purpose of the panels but it was instead to understand some perspectives of knowledge users and determine how they complemented the literature synthesis. Five reference groups were agreed and an expert panel member was identified to lead on a group. |
