# Supplementary material for: What are the mechanisms that support healthcare professionals to adopt assisted decision-making practice? A rapid realist review
Source: BMC Health Serv Res. 2019 Dec 12;19:960. doi: 10.1186/s12913-019-4802-x (PMC6909502; doi:10.1186/s12913-019-4802-x)
Supplement: Supplementary file 3 — Additional file 3. Rapid Realist Review: Literature screening process. [file 12913_2019_4802_MOESM3_ESM.docx]

**Additional file 3:**

**Rapid Realist Review: Literature screening process**

**Round 1: Title and abstract screening criteria**

| ***RRR Inclusion*** |
| --- |
| - Research papers on social/health care contexts and the perspective of healthcare professionals or patients/public have generated the data. - Research papers on care planning or care decision-making. |
| ***RRR Exclusion*** |
| - Perspective or theoretical papers. - Clinical decision-making without any interactive component with patients/public. |

**Round 2: Title and abstract screening criteria**

| ***RRR Inclusion*** |
| --- |
| - Papers which discuss shared decision-making or assisted/supported decision-making between healthcare professionals (including allied HCPs) and the following cohort of patients/public/participants experiencing any form of dementia or delirium, neuro-degenerative conditions which are impairing capacity temporarily or indefinitely (for example Parkinson’s disease, Huntington’s disease, motor neuron disease), mental health conditions or psychosis which are affecting their capacity, intellectual disabilities, loss of decision making capacity (for example those in a coma, on life support, under sedation, end of life etc.) - Papers which are discussing shared decision-making between healthcare professionals (including allied HCPs) and patients/public which refer to and discuss assisting decision-making capacity or competency - Papers which are discussing advanced care planning with patients/public who meet the criteria in inclusion point one above (or who are planning for the event they do meet those criteria). - Papers which are discussing shared/assisted decision-making between HCPs and surrogate decision-makers where a patient is found to have lost capacity. |
| ***RRR Exclusion*** |
| The following papers are excluded if they   - solely measure the prevalence of shared decision-making in different health contexts - investigate the association between health literacy and engagement in shared decision making. - investigate decision-making about participation in clinical trials. - developing and/or validating psychometric scales for measuring shared decision-making, decision-making generally, health literacy or treatment experience. - focus on euthanasia or physician-assisted suicide. - focus on maternity decision-making. - focus on paediatric decision-making – even if it is proxy or surrogate decision-making - focus on a general exploration of the different communication styles of healthcare professionals where they do not reference shared/assisted decision-making with those cohorts listed in inclusion point 1. |
