# Supplementary material for: What are the mechanisms that support healthcare professionals to adopt assisted decision-making practice? A rapid realist review
Source: BMC Health Serv Res. 2019 Dec 12;19:960. doi: 10.1186/s12913-019-4802-x (PMC6909502; doi:10.1186/s12913-019-4802-x)
Supplement: Supplementary file 4 — Additional file 4. Literature Synthesis Extract Template. [file 12913_2019_4802_MOESM4_ESM.docx]

**Additional file 4:**

**Literature Synthesis Extract Template**

The extraction template is informed by the Behaviour Change Wheel nine intervention functions and seven policy categories. It seeks to capture in a systematic way the range of interventions/mechanisms and policy initiatives that support healthcare professionals adopt ADM into practice (1).

**Behaviour Change Wheel descriptors**

| **Policies category**  *Actions on the part of responsible authorities that enable or support interventions*.   1. **Communication/marketing**: Using print, electronic, telephonic, or broadcast media. 2. **Guidelines:** Creating documents that recommend or mandate practice. This includes all changes to service provision. 3. **Fiscal measures:** Using the tax system to reduce or increase the financial cost. 4. **Regulation:** Establishing rules or principles of behaviour or practice. 5. **Legislation:** Making or changing laws. 6. **Environmental/social planning:** Designing and/or controlling the physical or social environment. 7. **Service provision:** Delivering a service.   Source: Michie S, Atkins L, West RT. The behaviour change wheel: a guide to designing interventions. Great Britain: Silverback Publishing; 2014 2014. |
| --- |
| **Interventions functions**  *Activities aimed at changing behaviour’.*   1. **Education:** Increasing knowledge or understanding. 2. **Persuasion:** Using communication to induce positive or negative feelings or to stimulate action. 3. **Incentivisation:** Creating an expectation of reward. 4. **Coercion:** Creating an expectation of punishment or cost. 5. **Training:** Imparting skills. 6. **Restriction:** Using rules to reduce the opportunity to engage in the target behaviour (or to increase the target behaviour by reducing the opportunity to engage in competing behaviours). 7. **Environmental restructuring:** Changing the physical or social context. 8. **Modelling:** Providing an example for people to aspire to or imitate. 9. **Enablement:** Increasing means/reducing barriers to increase capability (beyond education and training) or opportunity (beyond environmental restructuring).   Source: Michie S, Atkins L, West RT. The behaviour change wheel: a guide to designing interventions. Great Britain: Silverback Publishing; 2014 2014. |


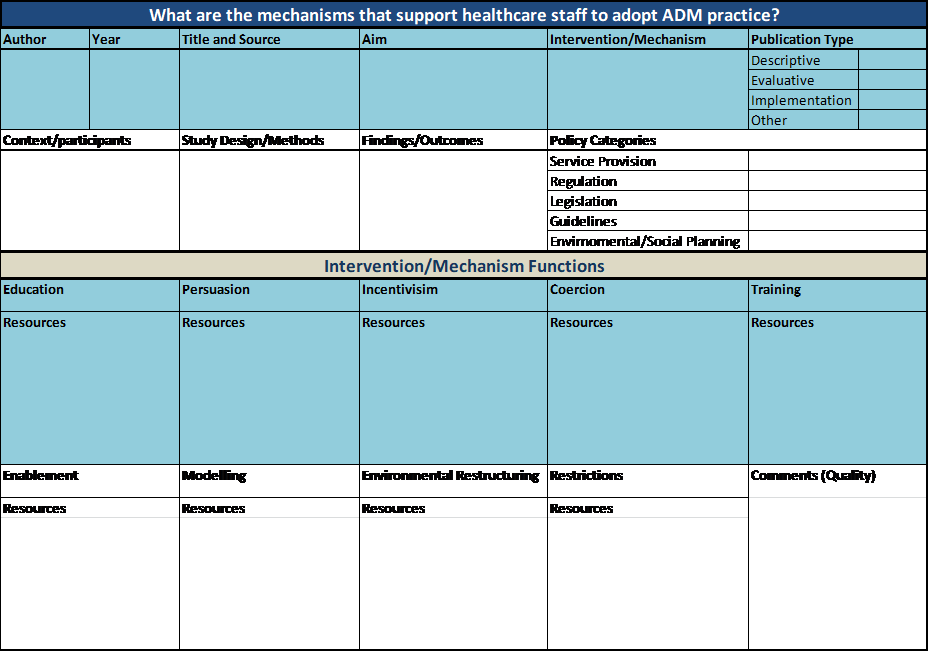


1. Michie S, Atkins L, West RT. The behaviour change wheel: a guide to designing interventions. Great Britain: Silverback Publishing; 2014 2014.
