# Supplementary material for: What are the mechanisms that support healthcare professionals to adopt assisted decision-making practice? A rapid realist review
Source: BMC Health Serv Res. 2019 Dec 12;19:960. doi: 10.1186/s12913-019-4802-x (PMC6909502; doi:10.1186/s12913-019-4802-x)
Supplement: Supplementary file 5 — Additional file 5. Programme Theories (PTs) Process- Phase 1-3. [file 12913_2019_4802_MOESM5_ESM.docx]

**Additional file 5: Programme Theories (PTs) Process- Phase 1-3**

Programme Theories (PTs) Process- concept map

The Rapid Realist Review seeks to identify programme theories to provide guidance on the implementation of Assisted Decision Making in the Irish healthcare system. Programme theories can be defined as the set of assumptions of key stakeholders that explain how they expect an intervention might achieve its objectives.

**
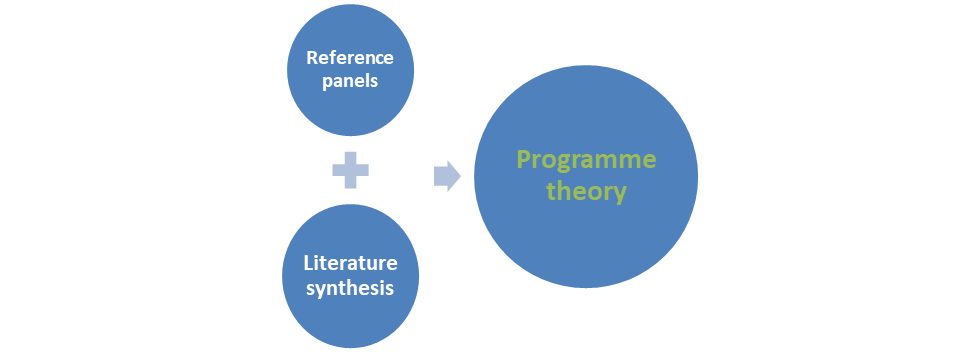
**

**Phase 1: Reference panels**

The summary notes from the five reference panels are represented here. After five reference panels, the RRR team held a consensus meeting (DOD, ÉNS, FF, CD, SD) and reviewed summary notes and agreed the key themes emerging of importance for each representative group. The main themes were represented as Initial Programme Theories (IPTs) to answer the question ‘What are the mechanisms that support healthcare professionals to adopt Assisted Decision Making?’

**Phase 2:**

The IPTs were later refined and expanded with the literature.

**Phase 3:**

The final PTs were reviewed and refined by the expert panel, and the final PTs was confirmed as represented in the main body of this paper.

**Phase 1:**

**Reference Panels Summary notes**

| **Alzheimer’s Society Ireland (ASI)**  **Grey literature provided.**  **Key issues in relation to ADM Capacity Act** |
| --- |
| **Key issues raised by people with dementia**   1. **Complexity of Dementia**   Dementia follows a predictable path in terms of the continued deterioration of a person's ability to make decisions and carry out everyday tasks. In the early to mid-stages, there may be considerable fluctuation in their ability. Time of day, familiar or unfamiliar setting, means of communication, physical health and other factors may all impact negatively or positively on the ability of a person with dementia to exercise capacity. People with dementia are concerned that this is understood by Health and Social Care Professionals (HSCPs).  There is considerable agreement among people with dementia that they are vulnerable, even before diagnosis and indeed from the point of diagnosis. They are vulnerable when living on their own and also in nursing homes, as people providing care are not always monitored. People with dementia may not be able to speak up, express need or ask not to be treated in a certain way. Having limited capacity means one is vulnerable. Also, people with dementia frequently have co-morbidity and multiple medical conditions. The nature of their condition and dependency on others for care may make them particularly vulnerable to abuse and neglect.   1. **Autonomy, Dignity and Decision-Making**   Ensuring that a person with dementia has the autonomy to make decisions and that they are central to decision-making is a crucial concern for people with dementia. Although it is obligatory to protect a person with dementia from seriously harmful consequences, it is equally obligatory to respect his/her role in decision-making. This is echoed by people with dementia themselves where the Irish Charter of Rights for People with Dementia states that people with dementia should be allowed to exercise their remaining capacities for choice, consistent with their values, wishes, will and preferences and cultural expectations (Irish Dementia Working Group and ASI, 2016). This enables them to be independent and to live with dignity. In making decisions it is important to ensure that a person is involved, capacity is assumed, and a decision is based on a person's values, wishes and will and preferences.   1. ***Helping a Person make Decisions***   The system of assisted decision making should be flexible to allow people with dementia the time and support they need to understand, consider and communicate a decision. Practical steps should be taken to help a person with dementia make a decision. This can include the following examples:   - - Establishing a time of day at which a person functions best and approaching them at that time.   - Establishing the environment in which the person functions best and approaching them in that environment.   - Establishing the people with whom the person communicates best and involving them in the communication process.   - Explaining written information orally, or writing oral information down in simple form to enable retention of information.   - Discussing the same issue on several different occasions to establish whether the person's view remains consistent.  1. **Accountability**   People with dementia believe that bodies and individuals responsible for their care and treatment should be accountable for the respect, protection and fulfilment of their human rights and adequate steps should be adopted to ensure this is the case.   1. **Training and Trust**   People with dementia are of the view that appropriate training on dementia among HSCPs is crucial. Clinicians and other HSCPs should be aware that the mental capacity of a person with dementia can change over time both in the short and long term. This means that HSCPs have a significant responsibility to support people with dementia to make person-centred decisions as their mental capacity changes. People with dementia feel it is important that they can trust HSCPs, and the latter will require in-depth understanding of how this ADM legislation impacts on people with dementia, what their responsibilities are, on appropriate procedure, and how they can support the latter to make a person-centred choice, centring on respect for identity and dignity.   1. **Use of Medications**   People with dementia have expressed serious concern around the use of antipsychotic drugs, as have carers. The decision to use antipsychotic drugs in the management of behavioural and psychological symptoms of people with dementia needs to be considered with extreme caution, and people with dementia and carers need to be involved in these decisions. |
| **Rehabilitation setting**  **Workshop with 13 members of the Healthcare Professionals (HCPs)**  **Presentation of case studies where ADM was relevant** |
| *The following key themes emerged :*   1. **ADM is influenced by the context of the clinical setting, purpose of care and patient group**   ADM in a rehabilitation setting requires making decisions about long term care. The assessment of functional capacity is within the context of emerging capacity rather than decreasing capacity.  Clinician experiences of supporting ADM is that it raises ethical dilemmas   1. ***Culture & Leadership***  - The importance of organisational commitment to resource, promote and sustain ADM within the practice is necessary. Both senior management and front line staff are engaged. - Policy influences include the HSE consent policy, policy ADM legislation, Charter Human Rights - An essential resource is the availability of legal advice- *Ireland culture in health system risk-averse*  1. **Importance of a team-based model within ADM service provision.**  - Team collaboration is a critical model for promoting ADM. Multidisciplinary team involvement – a collaborative approach using the combined expertise of all HCPs. - Based on case experience, staff expressed that the quality of the interdisciplinary teams as a functioning unit influence the outcome. Positive outcomes through team-based approaches generate an intrinsic reward and sense of satisfaction with care. Decisional conflict does occur among teams at times.  1. **Resources**  - Reality and resources within the health and social system in conflict with ADM - Negative consequences for the team and patient are that assisted decision, particularly about care planning, is not supported by resources and services within the Irish healthcare system. Dissatisfaction in terms of decisional burden – uncertainty and concern around honouring patient preferences and decisions. Feel choices are limited (i.e. social influences, housing crisis, wheelchair accessibility) - Resource intensive in terms of time and work planning.  1. **Involvement of Family involvement in care planning**  - System hugely relies on family yet it can be emotional dialogue at times with difficult conversations. Family dynamics can be present and oftentimes family members are shocked that they do not have decision-making power.   **Mechanisms that might support staff**   - Communicative systems to suit patient needs. - Case review learning for teams/organisation (Fear of liability with staff). - Consultation forum might be valuable for shared reflection and learning. - Documentation/proformas – valuable as it would scaffold process. - CPD Training Certificate - efficacy training -Support ongoing training for staff to take ‘unsafe decisions. Need for educating patients and family members on the legislation. Skills for team-based social relations |
| **Acute Care setting**  **Workshop with HCPs working in acute care setting** |
| **The following were the main issues raised by health care professionals in relation to ADM Capacity Act.**  **1** **HCPs main concerns about the ADM legislation and practical issues.**   - The conflict between autonomy as a legal principle and beneficence as an ethical principle within medicine. - The removal of ‘best interest’ out of the legislation is a concern for health care professionals. The example provided by the potential for a delay in surgery and that a person may be progressing into a delirium. - Staff expressed the need for guidance on these practical issues. - Guidance on emergency surgery - Concern regarding –occasions of no capacity and no relatives   **2 Consequences of clinician-family relation and negative potential on the outcome**   - There was a generalised concern of how HCPs will be able to deal with families and negotiate the family concerns and family dynamics. Much of these concerns were based on their previous experience in these situations. For example, cases, where there is no consensus among family or the primary representative of the family, does not concur with the rest of the family.   **3** **Organisation level consequences**   - The concern of workload increasing and overburden on the medical system. - Concern around the capacity and capability of who has the autonomy to conduct FCA. - Recognition of the need to modify current work practices to distribute leadership and responsibility. - They were a question whether there would be panels for of HCP to assess the capacity. - Some of the social workers had practised ADM in the UK, but this is not current practice in SVUG - In terms of the roles and responsibilities to conduct an FA- for example, if a solicitor asks a consultant   **4**: **Support and guidance**   - HCPs need clarity on the process - At present, there is no training or code of practice. - SWs are not trained on capacity - AF reiterated that Allied HCP should be doing capacity- not for medical staff alone. - Questions about legal support within hospitals   **Policy/Intervention planning**   - Concern about Governance. AF: HCP have a duty to report co-decision maker - Questions on how speedy the process will be to process records Interventions to maximising capacity- this is currently in the HSE policy. - Considering ways to Intensify interventions to maximise capacity (Documentation – capacity assessment form standard)   **Health system resource limitations**   - Questions about the health system /service not having the resources and services to support the preferences of the decision of patients |
| **Public consultation Family Carers Ireland (FCI)**  **Workshop and discussion** |
| **Key Points from** Family Carers Ireland (FCI) on the key issues of the Assisted Decision-Making Act (2015) for family carers.  FCI welcome the legislation. Up to now families, who wanted to act in the best interests and support their loved one, have had to navigate several legal deficits. These deficits have forced them, through no fault of their own, to make decisions on behalf of their loved one without legal protection, support or guidance.  The welcome for legislation which attempts to address these legal deficits comes with a caution.   - The family carer is not mentioned in the text of the Act, yet FCI stresses that the Act is predicated upon families’ willingness to engage with and sustain it. This was particularly the case when it came to the role of decision-making supporters as well as powers of attorney; who would, for the most part, be family members. - Most family carers (as parents) would welcome the principle of minimum intervention in the Act whereby decision-making processes should be least restrictive of a person’s rights and freedoms. - One could argue, however, that there is an element of ‘best interests’ underpinning the desire to implement decision-making processes which are ‘least restrictive of a person’s rights and freedoms’ and are at the minimum level of intervention. - The issue of the mandating of ‘unwise decisions’ which are informed by ‘will and preferences’ rather than ‘best interests’ was raised as a concern and the need for the state to consider if they will assume responsibility for the unintended consequences of decisions. - The legal framework is silent on the extent to which family carers have an obligation to facilitate as distinct from accepting the will and preferences of the person they care for. “Does it follow that a person deemed capable of making a decision under the Act is also deemed capable of implementing it and living with the consequences?”   Three concerns that represent Family carer members include:   - potential costs for family carers and overcrowded courts - Potential for conflicts between the different decision-supporter roles - A fear that the state will use the individual’s ‘will and preferences’ to live at home as a basis to neglect services and supports to caring families |

**Summary of the reference panels**

**Initial programme theory**

| **Summary of nine Initial programme theory from the reference panel** | | | |
| --- | --- | --- | --- |
| **Context** | **Intervention Functions/**  **policy** | **Outcome** | **Initial Programme Theory** |
| **Legislation & codes of practice need to be adapted into the healthcare settings using setting specific policies** | Guidelines  Environment & Social Planning    Service provision | Assisted Decision Making is an accepted norm embedded in the healthcare organisation. | (IPT1) Organisations embed the ADM codes of practice through introducing setting (context)-specific policies to support ADM.  Policy initiatives should include clinical guidelines, environment and social planning and service provision required for ADM implementation. |
| **Organisations that have high functioning models of interdisciplinary teams delivering patient-centred care support ADM.** | Restructuring social context (work processes). | ADM work processes are embedded in models of person-centred care.  ADM work processes are embedded in high functioning interdisciplinary teams.  Interdisciplinary Schwartz wards (debriefing and reflection) facilitate healthcare professionals in practising effective ADM. | (IPT 2) Organisations with high functioning interdisciplinary teams with a commitment to person-centred care facilitate work processes that foster effective ADM practices.  (IPT 3) Organisations that embed supportive mechanisms for interdisciplinary teams can de-brief and learn through reflection about ADM. |
| **ADM requires organisations to embed an implementation model to support ADM** | Restructuring social context | Organisation utilise an appropriate model, i.e. Interdisciplinary learning collaborative or clinical micro-systems to engage healthcare teams in the implementation of ADM | (IPT 4 – Strictly related to IPT1)  Organisations that embed implementation models that facilitate senior and frontline multidisciplinary engagement foster more effective ADM implementation (i.e. collective leadership). |
| **Patients and family carers require support from healthcare organisations/healthcare professionals.** | Physical and social restructuring of the  Environment  Enablement | Healthcare organisations provide a service, i.e. ‘resource hub' and specialist experts to support patients and family carers to learn about the ADM. | See later IPT |
| **ADM legislation is complex and requires knowledge and understanding of its application to various healthcare settings and practices.** | Education  Enablement | Healthcare professions have appropriate knowledge & understanding of the ADM legislation to adopt ADM into their practice.  Patients and family carers have access to ADM resources and support within the healthcare system so they can gain appropriate knowledge and understanding of the ADM legislation to enable them to understand and engage in the ADM process. | (IPT5) Education and training programmes for all hospital staff using active mode learning facilitate the knowledge, understanding and the delivery/practice/implementation of the ADM legislation.  (IPT 6) Supporting patient and family carers in educational support programs will foster positive relationships in the ADM process.  (embed patients' and family members perspective on mandatory education and training programmes for staff). |
| **ADM is a complex intervention and requires healthcare professionals to have requisite skills** | Training –imparting skills in ADM competence  Enablement | ADM competency requires skills in  Inter-personal clinician /patient communication  Functional Capacity Assessment skills  Dementia training skills  Enabling tools, e.g. FCT, process documentation and assistive communication technologies can support the ADM process. | (IPT 7) Healthcare professionals require requisite all-around skills and supporting tools to develop competency in adopting and practising ADM (i.e. professional standards, code of ethics, interdisciplinary teamwork)  (IPT 8 – related to IPT 5) Healthcare professions that have dementia training develop competency in ADM with people with dementia. |
| **ADM requires positive clinician-patient & clinician –family carer relationships.** | Education  Training  Enablement | Healthcare professionals, patients and family carers have knowledge and understanding of the role of the co-decision maker in the ADM process.  Specialised communication skills enhance positive clinician – family relations. | (IPT 9) Patients and family carers with shared knowledge and understanding of the ADM legislation foster better clinical – family relationships and better outcomes for the patient in the ADM process. |

| **Phase 3: Refining and confirming PTs.**  **Expert panel meeting November 2018**  During the second meeting with the expert panel, we updated the members presenting the progress of PADMACS and asking them to provide us with suggestions about the four IPTs we created after the integration between the reference panels meetings and the literature review. The experts commented on and contributed to the refinement of the IPTs.  Here below the comments related to the PT: |
| --- |
| **PT1**   - ADM as a core principle of PCC - The concept of Advance Care Planning refers to full capacity – reflection about dropping “Advance” and just leaves “care planning.” - Interdisciplinary - all the HCPs, across the board |
| **PT2**   - Focus on staff support – system-wide commitment - The risk to end up as in England or Wales where they still work with “best interest” approach - “Relevant person” and “Person-centeredness” operative definitions needed, risk of tokenism - Need to enhance PT with some generic principles from Implementaiton science |
| **PT3**   - Add “interdisciplinary” to the PT statement - Medical council guidelines and Consent policy) leave a relevant grey area where medical decisions are arbitrary - Guidelines & supportive templates |
| **PT4**  **•** ADM skills should be embedded in the clinical training  • Public campaign endorsed by everyone |
