# Supplementary material for: What are the mechanisms that support healthcare professionals to adopt assisted decision-making practice? A rapid realist review
Source: BMC Health Serv Res. 2019 Dec 12;19:960. doi: 10.1186/s12913-019-4802-x (PMC6909502; doi:10.1186/s12913-019-4802-x)
Supplement: Supplementary file 6 — Additional file 6. Summary of literature for Review. [file 12913_2019_4802_MOESM6_ESM.docx]

**Additional file 6: Summary of literature for Review**

| **Authors & Year** | **Journal** | **Title** | **Country** | **PT** |
| --- | --- | --- | --- | --- |
| Aydin Er & Sehiralti  2014 | *Journal of Medical Ethics* | Comparing assessments of the decision-making competencies of psychiatric inpatients as provided by physicians, nurses, relatives and an assessment tool | Turkey | PT 2 |
| Black et al  2009 | *Journal of Aging and Health* | Surrogate decision-makers' understanding  of dementia patients' prior wishes for  end-of-life care | USA | PT 1 |
| Brazil et al  2015 | *BMC Palliative Care* | General practitioners perceptions on advance care planning for patients living with dementia | Northern Ireland | PT 1 & 3 |
| Brimblecombe et al. 2014 | *Internal Medical Journal* | The goals of the Patient Care project: Implementing a proactive approach to patient-centred decision-making | Australia | PT 1, 2 & 3 |
| Bronner et al  2016 | *BMC research notes* | Which medical and social decision topics are important after the early diagnosis of Alzheimer's disease from the perspectives of people with Alzheimer's disease, spouses and professionals | Germany | PT 1 |
| Bunn et al  2018 | *BMC Geriatrics* | Supporting shared decision making for older people with multiple health and social care needs: A realist synthesis | UK | PTs 1, 2, 3, 4 |
| Butterworth & Campbell  2014 | *British Journal of General Practice* | Older patients and their GPS: Shared decision making in enhancing trust | UK | PT 1,2 & 3 |
| Campbell et al  2013 | *American Journal of Psychiatric Rehabilitation* | The effect of CommonGround software and decision support centre | USA | PT 3 |
| Cartwright et al  2016 | *Palliative Medicine* | Palliative care and other physicians' knowledge, attitudes and practice related to the law on withholding /withdrawing life-sustaining treatment | Australia | PT 3 |
| Cairncross et al  2016 | *Cambridge Quarterly of Healthcare Ethics* | Assessing decision-making capacity in patients with communication impairments | Canada | PT 2 |
| Catic et al  2013 | *Journal of the American Geriatrics Society* | Preliminary data from advanced dementia consult service: integrating research, education, and clinical expertise | USA | PT 1 & 3 |
| Chiarchiaro et al  2017 | *American Journal of Respiratory and Critical Care Medicine* | Physicians rarely elicit critically ill patients' previously expressed treatment preferences in intensive care units | USA | PT 1 |
| Cogo et al  2016 | *Revista Brasileira de Enfermagem* | Challenges to implementation of advance directives of will in hospital practice | Brazil | PT 1 |
| Cox et al  2012 | *Critical Care Medicine* | Development and pilot testing of a decision aid for surrogates of patients with prolonged mechanical ventilation. | USA | PT 3 |
| De Vleminck et al  2014 | *PloS One* | Barriers to advance care planning in cancer, heart failure and dementia patients: A focus group study on general practitioners' views and experiences | Belgium | PT 1 |
| De Vleminck et al  2016 | *PLoS ONE* | How do general practitioners conceptualise advance care planning in their practice? A qualitative study | Belgium | PT 1 |
| De Vleminck et al  2016 | *BMC palliative care* | Development of a complex intervention to support the initiation of advance care planning by general practitioners in patients at risk of deteriorating or dying: a phase 0-1 study | Belgium | PT 1 |
| Donnelly et al. 2018 | *Dementia* | How are people with dementia involved in care planning and decision-making? An Irish social work perspective | Ireland | PT 1,2 & 3 |
| Eloranta et al 2014 | *International Journal of*  *Nursing*  *Practice* | Finnish nurses' perceptions of care of older patients | Finland | PT 3 |
| Escher et al  2015 | *Journal of Pain and Symptom Management* | Doctors' decisions when faced with  contradictory patient advance directives  and health care proxy opinion: a  randomized vignette  based study | Switzerland | PT 1 |
| Fetherstonhaugh et al  2016 | *Journal of Applied Gerontology* | “The red dress or the blue?" How do staff perceive that they support decision-making for people with dementia living in residential aged care facilities? | Australia | PT 2 & 3 |
| Fried et al  2010 | *Journal of the American Geriatrics Society* | Stages of change for the component behaviours of advance care planning | USA | PT 1 |
| Fritch et al  2013 | *Journal of Clinical Ethics* | Making decisions for hospitalised older adults: Ethical factors considered by family surrogates | USA | PT 1 |
| Gainer et al  2017 | *Medical Decision Making* | Toward optimal decision making among vulnerable patients referred for cardiac surgery: A qualitative analysis of patient and provider perspectives | Canada | PT 2 & 3 |
| Galambos et al  2016 | *Health & Social Work* | Analysis of advance directive documentation to support palliative care activities in nursing homes | Canada | PT 3 |
| Gigon et al  2015 | *Medicine (Baltimore)* | Advance directives and communication skills of prehospital physicians involved in the care of cardiovascular patients | Switzerland | PT 1 & 3 |
| Gigon et al  2015 | *Minerva Anestesiologica* | Swiss physicians' perspectives on advance directives in elective cardiovascular surgery | Switzerland | PT 1 & 3 |
| Gjerberg et al  2015 | *BMC Geriatrics* | End-of-life care communications and shared decision-making in Norwegian nursing  Homes. Experiences and perspectives of  patients and relatives | Norway | PT 1, 2 & 3 |
| Hadler et al  2016 | *Anesthesia & Analgesia Case Reports* | Advance directives and operating: Room for improvement? | USA | PT 1 |
| Hamann et al  2014 | *The International Journal of Social Psychology* | Effects of a question prompt sheet on active patient behaviour: An RCT with depressed outpatients | Germany | PT 3 |
| Hamann et al  2017 | *Social Psychiatry and Psychiatric Epidemiology* | Training patients with schizophrenia to share decisions with their psychiatrists: A randomised controlled trial | Germany | PT 3 |
| Holland et al  2013 | *International Journal of Clinical Practice* | Barriers to involving older people in their resuscitation decisions: The primary-secondary care mismatch highlights the potential role of general practitioners | England | PT 2 & 3 |
| Kwak et al  2016 | *Research in Gerontological Nursing* | Role of advance care planning in proxy decision making among individuals with dementia and their family caregivers | USA | PT 1, 2 & 3 |
| Larsen et al  2017 | *Dementia* | Processes of user participation among formal  and family caregivers in home-based care for persons with dementia | Norway | PT 4 |
| Livingston et al  2010 | *BMJ (Clinical research education)* | Making decisions for people with dementia who lack capacity: Qualitative study of family carers in the UK | London | PT 3 |
| Mann et al  2013 | *Journal of the American Geriatrics Society* | Do-not-hospitalize orders for individuals with advanced dementia: Healthcare proxies' perspectives | USA | PT 2 & 3 |
| Mariani et al  2017 | *Aging & Mental Health* | Shared decision-making in dementia care planning: Barriers and facilitators in two European countries | Italy & Netherlands | PT 2, 3 & 4 |
| McCarthy  2010 | *British Journal of Learning Disabilities* | Exercising choice and control. Women with learning disabilities and contraception | Kent  England | PT 3 |
| Murray et al  2016 | *Palliative Medicine* | Advance care planning in motor neuron disease: A qualitative study of caregiver perspectives | Australia | PT 2 & 3 |
| Poole et al  2014 | *BMC Geriatrics* | Going home? An ethnographic study of assessment of capacity and best interests in  people with dementia being discharged from  hospital | England | PT 2 |
| Robinson et al  2013 | *Palliative Medicine* | A qualitative study: Professionals’ experiences of advance care planning in dementia and palliative care, ‘A good idea in theory but …’ | England | PT 3 & 4 |
| Smebye et al  2012 | *BMC Health Services Research* | How do persons with dementia participate in  Is decision making related to health and daily care?  A multi-case study | Norway | PT 1 & 3 |
| Sulmasy  2017 | *Journal of Pain and Symptom Management* | The trial of ascertaining individual preferences for loved ones' role in end-of-life decisions (tailored) study: A randomised controlled trial to improve surrogate decision making | USA | PT 3 |
| Torke  2012 | *Journal of the American Geriatrics Society* | Communicating with clinicians: the experiences of surrogate decision-makers for hospitalised older adults | USA | PT 2 |
| Torke  2009 | *Journal of General Intern Medicine* | Physicians' experience with surrogate decision making for hospitalised adults | USA | PT 3 |
| Werner et al  2017 | *Patient Education & Counseling* | Principles of effective communication with patients who have intellectual disability among primary care physicians | Israel | PT 1 & 3 |
| Wilkinson et al  2013 | *Journal of Intellectual Disability Research* | Patient and provider views on the use of medical  services by women with intellectual disabilities | USA | PT 2 |
